# Stroke Survivors’ Personal Efficacy Beliefs and Outcome Expectations of Tai Chi Exercise: A Qualitative Descriptive Study

**DOI:** 10.3390/ijerph182413001

**Published:** 2021-12-09

**Authors:** Ruth Taylor-Piliae, Hanne Dolan, Aodet Yako

**Affiliations:** College of Nursing, University of Arizona, 1305 N. Martin, P.O. Box 210203, Tucson, AZ 85721-0203, USA; hannedolan@email.arizona.edu (H.D.); yako@email.arizona.edu (A.Y.)

**Keywords:** complex systems biology, focus groups, qualitative research, social cognitive theory, stroke rehabilitation, tai chi

## Abstract

Prior qualitative research conducted among stroke survivors to explore the potential benefits and challenges of participating in tai chi exercise during stroke recovery is limited to those without depression. A qualitative descriptive approach was used. Social Cognitive Theory and Complex Systems Biology provided the theoretical framework, with focus group interview data collected from stroke survivors after participation in a tai chi intervention. Due to COVID-19, the focus group interview was conducted via online video conferencing. Content analysis of the de-identified transcript was conducted with a-priori codes based on the theoretical framework and inductive codes that were added during the analysis process. Lincoln and Guba’s criteria were followed to ensure trustworthiness of the data. Community-dwelling stroke survivors (*n* = 7) participating in the focus group interviews were on average 68 years old, mainly retired (71%, *n* = 5), married women (57%, *n* = 4) with >13 years education (86%, *n* = 6). The three major themes were: personal efficacy beliefs, tai chi intervention active ingredients, and outcome expectations. Social Cognitive Theory underscored stroke survivors’ personal efficacy beliefs, behavior, and outcome expectations, while Complex Systems Biology highlighted the active ingredients of the tai chi intervention they experienced. Participation in the 8-week tai chi intervention led to perceived physical, mental, and social benefits post stroke.

## 1. Introduction

Globally, strokes are estimated to affect roughly 102 million people. There are an estimated 7.6 million stroke survivors (>20 years old) living in the United States (U.S.), with stroke prevalence increasing among both men and women with advancing age [1]. Between 2015 and 2035, total direct medical stroke-related expenses are projected to increase significantly, from $36.7 billion to $94.3 billion, with the majority of these costs coming from those 80 years of age and older. Strokes account for approximately one of every 19 deaths in the U.S. and are ranked as the fifth cause of death, with heart disease, cancer, chronic lower respiratory disease, and unintentional injuries/accidents as the top four leading causes of death, respectively. Moreover, strokes are a leading cause of serious long-term disability in the U.S., which is often compounded by post-stroke depression experienced by an estimated 2.5 to 5 million stroke survivors [1,2]. Despite the availability of pharmacotherapies and/or psychotherapies, post-stroke depression persists even 5–10 years post-stroke, reflecting limited treatment responses and/or adherence to this conventional care [3,4]. Thus, incorporating mind-body approaches alongside conventional care to manage post-stroke depression is an important step to address this problem [2,5].

Tai chi exercise is an established mind-body approach to improve health and well-being among stroke survivors, including those with depression [6,7,8,9,10]. Tai chi is a moderate-intensity exercise integrating physical movements, breathing training, and mindful awareness during practice [11]. Prior tai chi research studies conducted among adults with cardiovascular disease, including stroke survivors, have reported that participants had greater exercise self-efficacy following a tai chi exercise program [12,13,14]. In addition to greater self-efficacy, these participants reported having greater social support, being empowered with better self-awareness and stress management after participating in a tai chi exercise program [13,14].

### 1.1 Theoretical Framework

**Figure 1:**
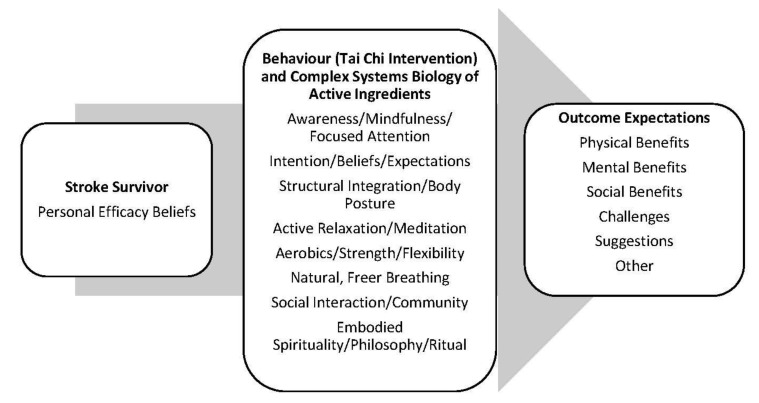
Theoretical Framework.

Social Cognitive Theory [15] provided the theoretical framework to guide this study, to examine stroke survivors’ personal efficacy beliefs, the tai chi intervention (behavior), and outcome expectations. It is well established that personal efficacy beliefs and outcome expectations influence the performance of an exercise behavior, such as tai chi [16,17,18,19]. Personal efficacy beliefs refer to the confidence an individual has to perform a specific behavior, while outcome expectations refer to the confidence that performing a specific behavior will lead to a desired effect. In addition, to allow for tailored tai chi exercise interventions to fit specific populations, the eight interdependent active ingredients of the tai chi intervention, as outlined in the Harvard Medical School Guide to Tai Chi [11], were examined to allow for a deeper understanding of this exercise behavior (see Figure 1).

Prior qualitative research conducted among stroke survivors to explore the potential benefits and challenges of participating in tai chi exercise during stroke recovery is limited to those without depression [14,20,21]. However, those findings indicated that stroke survivors perceive tai chi as being beneficial for improving their physical abilities, psychosocial well-being, and greater confidence in performing activities of daily living. Collectively, these stroke survivors shared that tai chi exercise was enjoyable and provided social support with new friendships developed during the classes. They reported few, if any, challenges associated with participating in tai chi exercise as part of their stroke recovery [14,20,21]. It is unknown if post-stroke depression would alter these perceptions of tai chi exercise, what personal efficacy beliefs, besides exercise self-efficacy, are evident; or how stroke survivors view outcome expectations of tai chi. Thus, the purpose of this study was to provide an opportunity for community-dwelling stroke survivors with depressive symptoms to describe their experience of being in the tai chi intervention, including any benefits or challenges they had, and to offer any suggestions for improving how the tai chi classes were structured.

## 2. Methods

This study used qualitative descriptive methodology [22,23]. The purpose of qualitative descriptive methodology is to describe participants’ experiences and perceptions of a phenomenon. The methodology can provide rich descriptions of an experience, while the analysis stays close to the data [22,24]. Qualitative descriptive methodology can provide novel insight into how stroke survivors’ struggling with depressive symptoms experience participating in a tai chi intervention, and this insight is foundational for modifying or adapting tai chi interventions for specific populations of interest. Focus groups is a data collection methodology where a small group of participants are engaged in an informal discussion about a topic or an experience [25]. This study is reported in accordance with the Consolidated criteria for Reporting Qualitative research (COREQ) [26] and conforms with the principles outlined in the Declaration of Helsinki.

### 2.1. Participant Selection

Purposive sampling using opportunistic criteria [27] was used to enroll community-dwelling stroke survivors with depressive symptoms (Patient Health Questionnaire-9, score >5) [28] participating in a pre–post intervention study examining the feasibility of tai chi for stroke survivors with depressive symptoms. The tai chi intervention protocol used in the pre–post intervention study included correct body preparation, standing meditation and 24 basic movements from the Classic Wu style. Additionally, the eight active ingredients of tai chi, as outlined in the Harvard Medical School Guide to Tai Chi [11], were incorporated during the classes. All participants in the pre–post intervention study were invited by the principal investigator (first author) to participate in the focus group interview to share their experiences of being in the tai chi intervention. Approval to conduct the study was obtained from the Institutional Review Board at the University of Arizona. A total of nine stroke survivors (82%) in the feasibility study agreed to participate in the focus group interview and provided written informed consent prior to data collection. Recruitment for the feasibility study has been described elsewhere [29]. However, two participants were unable to join the focus group interview due to technical challenges (*n* = 1) or a medical appointment (*n* = 1). Thus, a total of seven community-dwelling stroke survivors (*n* = 7) participated in this qualitative study.

### 2.2. Setting

The participants in this focus group had a pre-existing relationship and the shared experience of participating together in a series of in-person, group-based tai chi intervention classes for one hour, three times per week, for eight weeks [29]. Participants were informed about the purpose of the focus group during the consent process. A key feature of focus group interviews is the potential to analyze what the informal interactions reveal about the participants’ collective sense of the individual experiences [25].

The focus group interview took place and was audio-recorded over Zoom Video Conferencing [30] since in-person interviews were not an option due to the COVID-19 pandemic [31]. Each participant was contacted by phone two days prior to the focus group interview by a research assistant (AY), who assisted the participants in setting up and testing Zoom on either their phone, computer, or tablet. This is recommended for qualitative research with video conferencing as the data collection format [32]. The benefit of using Zoom for qualitative interviews is that the participants can stay in their own homes, without having to travel to an interview location [31,33].

### 2.3. Data Collection

An interview guide was developed based on a literature review and evaluated by the study team prior to conducting the focus group. The first author, the principal investigator of the study, was the moderator during the focus group interview. The moderator ensured that all participants shared their experiences, including those with mild aphasia. The research assistant (AY) was also present during the focus group interview, took field notes, and was available to assist with any potential technical difficulties. This process ensured that all participants were able to follow the entire online video-conferencing focus group interview. The moderator began the focus group interview as follows: “*Now that you have completed the tai chi intervention for stroke survivors, we are interested in knowing your thoughts and ideas about participating*”. Then, the moderator posed five different questions, providing all participants the opportunity to respond. These questions were:

1. What was it like to practice tai chi for one hour, three times a week?
2. Did you notice any benefits?
3. Did you experience any challenges?
4. What suggestions do you have for improving how the tai chi classes are taught?
5. Do you have any final comments you would like to share?

The focus group interview was audio-recorded, then transcribed using an online transcription service (www.rev.com (accessed on 5 May 2020)). Once the focus group interview was transcribed, all personal identifiers were removed by the first author. During the focus group interview, field notes were recorded by the research assistant (AY) to compare with the interview transcript to ensure no content was missing. There was one focus group interview, which was 55 min in duration.

### 2.4. Data Analysis

The interview transcript was de-identified prior to uploading and analyzing in Dedoose, a secure, online qualitative software analysis platform [34]. Analysis of focus group interview transcripts is centered around the group perspective and areas of agreement and controversy. Thus, the quotes are from multiple participants as opposed to individual participants [35]. The focus is on group dynamics and interactions and not individual perspectives [25]. Content analysis of the transcript was conducted using both a deductive and an inductive coding procedure. A-priori codes were developed based on the theoretical framework (see Table 1), and inductive codes were added in the analysis process [36]. The transcripts were coded independently by the first and the second authors. The code applications were discussed, and consensus was reached.

Trustworthiness for this project was ensured using the criteria described by Lincoln and Guba [37]. Credibility was established through member-checking during the interview and analysis triangulation, where the two authors (RTP and HD) independently analyzed the transcript and then reached consensus about findings. Transferability was ensured by the thorough description of the findings and relating the findings to what was found in similar studies. Dependability and confirmability were ensured through the analysis process where the findings were discussed and consensus was reached, and a thorough audit trail was maintained.

## 3. Results

### 3.1. Description of Study Sample

The community-dwelling stroke survivors (*n* = 7) with depressive symptoms (PHQ-9 > 5) [28] who participated in the focus group interviews after participating in an eight-week tai chi intervention study were on average 68 years old, mainly retired (71%, *n* = 5), married women (57%, *n* = 4) with >13 years education (86%, *n* = 6). These participants were on average 11 months post stroke (range = 4–20 months), with 43% (*n* = 3) of these participants taking anti-depressant medications. The majority completed stroke rehabilitation (71%, *n* = 5) and reported having an ischemic stroke (71%, *n* = 5) with hemiparesis (57%, *n* = 4) but were able to walk 15 feet without assistance (86%, *n* = 6). Mild aphasia was only reported by one participant, but this participant was still able to share their experiences providing shorter explanations/responses.

### 3.2. Interview Findings

Overall, the participants’ experiences using Zoom for the focus group interview were good. All participants were able to sign in and participate on time. Participants would often make sure they had been heard, but there were no technological issues during the interview. The participants described and discussed their experiences of participating in the tai chi exercise intervention. The transcript was analyzed based on the theoretical framework, and the major findings were summarized into the three major themes: personal efficacy beliefs, tai chi intervention/active ingredients, and outcome expectations (see Figure 1). In particular, the participants discussed how they enjoyed the tai chi exercise classes and that tai chi benefitted them physically, mentally, and socially.

### 3.3. Personal Efficacy Beliefs

Personal efficacy beliefs are understood as how individuals process and integrate different sources of information regarding their capability to perform a certain action, and their behavior is related to this information [15]. The tai chi intervention expanded the participants’ “personal efficacy beliefs.” They described how prior to the intervention, they were unsure about their capability for not only physically doing the tai chi movements but if they would be able to participate in the intervention on a regular basis:

“The schedule was good, but I got into it mainly to find out whether or not I could show up three days a week and was glad to be able to do it most of the time.” (Participant #108)

“A long time before I signed up, I thought, oh, three times a week, the terrible word, the bad E word, which is exercising, but that was helpful to train me to make sure I at least did it three times a week and kept going with it. So, very good.” (Participant #110)

### 3.4. Tai Chi Intervention/Active Ingredients

#### 3.4.1. Awareness, Mindfulness, and Focused Attention

All of the active ingredients of the tai chi intervention were evident in the participants’ experiences, apart from “natural freer breathing”, which the participants did not discuss or describe. First, the participants described how “awareness, mindfulness, and focused attention” was beneficial for them not only regarding the tai chi intervention but regarding their rehabilitation in general. The participants experienced physical challenges from their stroke, and the awareness and focused attention lessened their frustrations and improved their physical abilities:

“The other thing we started out with each session, which I thought was fantastic, was that meditative element. We would come in and we would have to do some real meditative moments and focus on gratitude, focus on positivity. I thought that was just so beautiful, no matter what we were going to do next, catwalk or all the other physical challenges, I thought it was a great discipline, mental discipline, to just get us in a mental mode, to be not just receptive but like I say, so positive.” (Participant #103)

#### 3.4.2. Intention, Beliefs, and Expectations

The active ingredient of “intention, beliefs, and expectations” was evident when participants described how they made adjustments related to their rehabilitation and recovering from a stroke. They learned to have more patience with themselves and their progress, which lessened their frustrations:

“I was finding frustrations in some of the small tasks that I was relearning how to do post-stroke. I think that the meditative aspect of the tai chi and what I was doing, the patience that was required to learn the movements and let the balance come, helped me in just small things around the house that I was very, very frustrated that I was not able to do.” (Participant #101).

#### 3.4.3. Structural Integration and Body Posture

The participants expressed how the tai chi movements were an extension of other kinds of rehabilitation. The participants noticed the benefit of “structural integration and body posture” focusing on the specific aspects of the tai chi movements that improved their balance and coordination. This moved them forward in their recovery process:

“The tai chi was a combination of something that helped, something that was new and different, and also as we had discussed, during the actual practice, was that it forced the mind-body coordination to kick in, which from my therapy was something that I felt was key to making progress in the recovery process.” (Participant #101)

#### 3.4.4. Active Relaxation and Meditation

The participants discussed how the tai chi classes had benefitted them in the ability to mentally put frustrations aside to “actively relax and meditate” and get their body and mind ready for what they wanted to do:

“It’s such a good thing to learn, to just, no matter what’s going on, put that aside, focus on the positivity. It was a great component of the whole thing. I thought also, waking up the chi, up and down the body. I mean, that was also something I learned. …Meditatively, just learning about how we get our body ready for something, not just our body and our mind. Very powerful.” (Participant #103)

“But I think that the tai chi and all that went into it really helped me to be calmer, more accepting and not get so upset with myself with what I wasn’t able to do. Rather, I’d concentrate on what I could do and how I needed to adapt to be able to do it. As I say, that meditative thought process with the tai chi, I think was the key to that.” (Participant #101)

#### 3.4.5. Aerobics, Strengthening, and Flexibility

Some participants also reported that the tai chi intervention helped them improve their “aerobic endurance, strength, and flexibility.” This was experienced both outside of class and during the tai chi classes as well:

“Most of the benefit for me, besides the actual physical benefit of things that we had talked about, such as balance and coordination, for me it was an extension of the therapy that I had already been doing.” (Participant #101)

#### 3.4.6. Social Interaction and Community

The aspect of the tai chi intervention that the participants discussed the most and described as the most important benefit was the “social interaction and community.” They described the camaraderie among the participants that developed throughout the tai chi classes, and this kept the participants coming back. They enjoyed the positive social connections within the group and the support they received from each other:

“I think it was just wonderful to be with you guys. This group of really dedicated people, all of us in this together really wanting to learn. There was such a feeling of camaraderie. There was such a feeling that we were helping each other or rooting for each other. That’s so powerful. That’s a remarkable thing.” (Participant #103)

This social support within the group became evident during the focus group interview when one participant described feeling bad about not being able to do all the movements and holding the other participants back. When this concern was voiced, other participants expressed how this was not noticed, only how the participant was so positive, worked hard, and motivated the whole group. This shows the significance of the social aspect within these tai chi exercise group-based classes:

“I never felt that you were slower or less capable, quite the opposite. I felt you were one of the most positive people and contributed so much to that group dynamic that was so, so positive and powerful. All of you were terrific and full of good energy.” (Participant #103)

The participants valued the social support and friendships that kept them coming to class, and the participants missed the group after the conclusion of the tai chi intervention:

“I think I had a great time. I enjoyed the participants, everybody. I miss going there. And the CDs are good, or DVDs are good, but it’s not the same as to have the group with you.” (Participant #107)

“I think not only we benefit from it, but potentially made some really good friends with, I guess situations, sharing the situations.” (Participant #110)

#### 3.4.7. Embodied Spirituality, Philosophy, and Ritual

Only a few participants commented on the “embodied spirituality, philosophy, and ritual” of tai chi. They mainly had more questions than answers but were curious to know more:

“I think everything that was done and the way it was done was really brilliant and fantastic, I would have loved at the very beginning of this whole program to get a little more information about tai chi. What is the history of this discipline? For what purposes was it developed?” (Participant #103)

### 3.5. Outcome Expectations

#### 3.5.1. Physical Benefits

The themes within the outcome expectations were the physical, mental, and social benefits the participants experienced as well as challenges they encountered, their suggestions for improvements of the tai chi classes, and other thoughts they wanted to share. The participants discussed how they all felt the “physical benefits” of the improvement in their balance. They described how tai chi helped them to be able to do daily activities again, such as getting dressed on their own. The participants discussed how they had balance problems and how this is a common and frustrating problem for stroke survivors. They noticed their improvement in balance both in themselves and each other:

“I found where my limits are today and how to test them, and the tai chi was very beneficial in that and finding my new limitations for balance and just like, put your pants on one leg at a time, it feels good to be able to do things like that again. “(Participant #108)

The participants also expressed how they enjoyed the “physical benefits” of focusing on the whole body as opposed to training each extremity separately. This aspect moved them forward in their rehabilitation:

“I go to physical therapy. They have one person that does the arm and then another person that does the leg. I think doing both of them together really helped.” (Participant #106)

#### 3.5.2. Mental Benefits

The participants noticed the “mental benefits” of the tai chi exercises, such as how focusing holistically on the body, mind, and spirit improved their mental state. They discussed how they enjoyed learning something new. This group was very eager to learn a new form of exercise, which inspired and motivated them in their rehabilitation process:

“We were all struggling with some brain-based deficits, each one our own, but some that we have in common. I think it was definitely very stimulating to our healing because you have to learn something totally new. To learn how to move our bodies in different ways. To follow a different kind of sequence than anything we’ve done before. The learning, I think was very, very positive.” (Participant #103)

“Okay. The mental exercise was great because I didn’t realize having a stroke, how your brain works differently and just the repetition of turning this way and that way was as valuable as the exercise itself to put that in a memory bank where you start doing that automatic and go on to the next step and train your mind to do one step at a time, I thought was excellent.” (Participant #110)

#### 3.5.3. Social Benefits

These participants reported several “social benefits”, such as enjoyment of the tai chi classes and camaraderie with the other stroke survivors attending the same class:

“I enjoyed it very much. Even though my limitations with walking were so limited, I got a lot out of it. I enjoyed the group. I enjoyed the structure that it added to my day, to my weekly planning.” (Participant #102)

#### 3.5.4. Challenges

Despite reported physical, mental, and social benefits, for some participants, the tai chi classes were “challenging” physically:

“It was some days exhausting for me. I have to say that I would be physically challenged by doing that. I didn’t think I would be, but I put in the effort to do things even though… Sometimes I just had to sit out.” (Participant #102)

“I’m pretty much agreeing with everybody else. It was challenging, but not in any unusual way. It was good to have the challenge and to be pushed but not pushed over.” (Participant #108)

A few participants experienced “challenges” related to travel and transportation, as some were dependent on others to travel to the classes:

“Well, I think it’s isolated with us being all over the map to have a tai chi class.” (Participant #110)

“Maybe if tai chi was closer and more of something I can go to, it would be easier for me.” (Participant #106)

#### 3.5.5. Suggestions

Participants provided several “suggestions” related to the frequency of the tai chi classes, along with other opportunities for doing tai chi:

“I think that the schedule was just about right. I think that one hour three times per week seemed very good to be able to reinforce what we were doing. Sometimes it seemed like, well, I kind of can’t wait for it to be over because of other issues. But at the same time, it seemed as though that was necessary. Because I think maybe once a week or only two days a week, would not be enough to be able to get the overall benefit and maybe progress in terms of what you were doing because you wouldn’t get the reinforcement that you need.” (Participant #101)

“The only thing I was thinking about is if it would be doable for the caretakers at home to join in and learn the ropes also. I’ve had experience with caretaking and that is just as daunting mentally and physical as the other. I’m thinking a team situation, if it’s your spouse or someone living with you, would be good.” (Participant #110)

#### 3.5.6. Other Outcomes

Participants also related “other outcomes”, including their experiences learning tai chi, which is a martial art. They also shared that they had better sleep because of participating in the tai chi classes:

“I loved learning about a new form of martial arts, something I had never done before. I was always intrigued when people talked about tai chi, but it was wonderful to have an opportunity to actually get in there and try something new and different and fascinating.” (Participant #103)

“I thought it was good for me, the physical exertion. I found that I slept better on those days. I think that the physical challenge for me doing that for one hour was good.” (Participant #102)

## 4. Discussion

In this qualitative study, Social Cognitive Theory [15] underscored stroke survivors’ personal efficacy beliefs, behavior, and outcome expectation, while Complex Systems Biology highlighted the active ingredients of the tai chi intervention they experienced [11]. This group of stroke survivors experiencing depressive symptoms reported that participation in the eight-week tai chi intervention led to perceived physical, mental, and social benefits. The three major themes identified were personal efficacy beliefs, tai chi intervention/active ingredients, and outcome expectations. 

For the theme personal efficacy beliefs, we found that most of the participants looked forward to the tai chi classes, and their personal efficacy beliefs increased when they discovered how their physical abilities improved. In addition, they were surprised and pleased about how their ability to do the tai chi movements improved each week. Our findings that these stroke survivors experienced greater personal efficacy beliefs after participating in tai chi is similar to findings from a prior qualitative study with participants reporting greater confidence in their body and their own abilities [20]. Moreover, a mixed methods study examining the feasibility of tai chi for stroke rehabilitation [14] reported significantly greater self-efficacy in performing activities of daily living post-intervention. This result was supported by qualitative data [14] indicating greater confidence in performing activities of daily living following the tai chi intervention.

The theme tai chi intervention/active ingredients is a new area of research that, to our knowledge, has only recently reported the perceptions of these active ingredients among community-dwelling older adults without a history of stroke [38]. Similar to this observational study [38], stroke survivors in our study experienced all of the tai chi intervention’s active ingredients, apart from “natural freer breathing”, which they did not describe. However, natural freer breathing was mentioned frequently in the tai chi classes [11,39]. Moreover, we found that the social interactions and sense of belonging among these stroke survivors was a key active ingredient with therapeutic value as in prior studies [14,20,21], which led to self-discovery [11,13,40]. Furthermore, participants related a collective sense of the shared experience during the focus group, which was illuminated during the focus group interactions. In a qualitive study conducted among adults with heart failure, themes of increased mindfulness and self-awareness, meditation, and relaxation were identified [13]. The active ingredients of tai chi exercise distinguishes it from traditional forms of aerobic exercise; thus, further research is needed to understand these ingredients and to design appropriate interventions for populations with specific needs [38].

For the theme outcome expectations, our results are similar to prior qualitative studies with stroke survivors reporting physical, mental, and social benefits after their participation in tai chi exercise despite having different functional abilities [14,20,21]. Moreover, the stroke survivors in these studies reported physical benefits, such as better balance and strength; mental benefits, such as hope and less depression; and social benefits, such as new friendships and greater social support [14,20,21]. These findings are similar those reported by the stroke survivors with depressive symptoms in this study. Tai chi may be especially beneficial for older adults with post-stroke depression by lessening depressive symptoms, and this important aspect warrants further investigation. In addition, some stroke survivors expressed a desire to continue practicing tai chi after completion of the research study due to the perceived benefits they gained [21] and, similar to our findings, stated that tai chi would be good way of integrating various skills learned during rehabilitation [21]. Similar to our findings, stroke survivors in these studies reported few challenges associated with attending the tai chi classes [20,21]. 

In this qualitative study, we were unable to conduct an in-person focus group for data collection, as originally planned, due to COVID-19. Instead, online video conferencing using Zoom provided a cost-effective and convenient alternative enabling all participants to see each other while providing password protection for confidentiality and securely audio-recording the interview [31,32,33]. Participants received assistance two days prior to the focus group in setting up and testing Zoom; however, a few participants still experienced minor technical difficulties, such as having their camera angle partially obscuring their face, challenges with turning the mute button off and on, or with microphone volume adjustments.

Focus groups often bring together a group of people with a shared experience to specifically to talk about that experience [25,35]. Since the study participants had been together in the tai chi classes for eight weeks, conducting a focus group interview was viewed as acceptable, as they all knew each other. They were readily able to share their individual experiences and beliefs about their participation in the tai chi intervention. This decreases some of the bias/limitations inherent with focus groups. However, in this study, we only conducted one focus group, which likely omitted the views and beliefs of other stroke survivors participating in tai chi exercise interventions. Finally, some of the participants had difficulty speaking due to stroke and were less likely to share their experiences during the focus group. However, all participants were encouraged to share their experiences and were provided with the opportunity to do so. 

## 5. Conclusions

In this qualitative study, community-dwelling stroke survivors with depressive symptoms described their experiences of being in a tai chi intervention, including any benefits or challenges they had, and offered suggestions for improving how the tai chi classes were structured. A focus group interview was conducted using online video conferencing due to COVID-19 but still enabled all participants to see each other. Social Cognitive Theory underscored stroke survivors’ personal efficacy beliefs, behavior, and outcome expectations, while Complex Systems Biology highlighted the active ingredients of tai chi intervention they experienced. Participation in the eight-week tai chi intervention led to perceived physical, mental, and social benefits post stroke.

## Figures and Tables

**Table 1:** A-Priori Deductive Codes and Sub-Codes.

A Priori Deductive Codes and Sub-Codes
Personal Efficacy Beliefs Tai Chi Intervention Active Ingredients
Awareness/Mindfulness/Focused Attention
Intention/Beliefs/Expectations
Structural Integration/Body Posture
Active Relaxation/Meditation
Aerobics/Strengthening/Flexibility
Natural Freer Breathing
Social Interaction/Community
Outcome Expectations
Physical benefits
Mental benefits
Social benefits
Challenges
Suggestions
Other

## Data Availability

The data presented in this study are available on request from the corresponding author. The data are not publicly available for privacy reasons.
